# Preliminary exploration of metagenomic sequencing for pathogenic identification in infectious uveitis

**DOI:** 10.1186/s12348-024-00449-3

**Published:** 2024-12-31

**Authors:** Jinxia Yu, Yane Gao, Hongsheng Bi, Youhua Zhang, Kai Tang, Dadong Guo, Xiaofeng Xie

**Affiliations:** 1 Department of Ophthalmology, Affiliated Eye Hospital of Shandong University of Traditional Chinese Medicine, Jinan, 250002 China; 2 Department of Ophthalmology, The Second Affiliated Hospital of Guizhou University of Traditional Chinese Medicine, Guiyang, 550000 China; 3 Shandong Provincial Key Laboratory of Integrated Traditional Chinese and Western Medicine for Prevention and Therapy of Ocular Diseases, Shandong Academy of Eye Disease Prevention and Therapy, Jinan, 250002 China; 4 Shandong University of Traditional Chinese Medicine, Jinan, 250014 China; 5 Medical College of Optometry and Ophthalmology, Shandong University of Traditional Chinese Medicine, Jinan, 250002 China

**Keywords:** Metagenomic sequencing, Infectious uveitis, Microorganism, Pathogen diagnosis

## Abstract

**Purpose:**

To evaluate the advantages and clinical utility of metagenomic sequencing (MGS) in diagnosing infectious uveitis pathogens.

**Methods:**

A retrospective study was conducted on 20 infectious uveitis patients (20 eyes) who received treatments at the Affiliated Eye Hospital of Shandong University of Traditional Chinese Medicine from May 2020 to February 2021. Anterior aqueous humor of the patients was collected and analyzed using MGS. Then, pathogenic microorganisms that cause uveitis were identified through bioinformatic analyses based on the sequencing data of MGS. Finally, the pathogens identified by MGS were verified using both enzyme-linked immune sorbent assay (ELISA) and quantitative PCR (qPCR).

**Results:**

MGS was used to detect viral pathogens in four patients, bacterial pathogens in two patients, and viral and bacterial pathogens in one patient. Among these seven subjects, five were verified by either ELISA or qPCR.

**Conclusions:**

MGS holds significant value and promising potential in diagnosing infectious uveitis pathogens. However, it cannot completely replace the traditional diagnostic techniques and still needs to be integrated with conventional methods to enhance the sensitivity and specificity of pathogen detection. As a pioneering technology, MGS will advance the field of pathogen diagnosis in infectious uveitis.

**Supplementary Information:**

The online version contains supplementary material available at 10.1186/s12348-024-00449-3.

Uveitis is one of the main causes of blindness, accounting for 10–15% of severe visual disturbances [1, 2]. Broadly, uveitis is classified into infectious and non-infectious types. The pathogens of infectious uveitis mainly include bacteria, fungi, viruses, and parasites, and the onset and development of infectious uveitis can be very rapid. Consequently, the prompt and accurate identification of causative pathogens is critical for effective management of infectious uveitis [3]. Currently, traditional diagnostic techniques rely on the isolation and purification culture of pathogenic microorganisms, but these approaches are limited because more than 99% of the pathogenic microorganisms cannot be cultured under standard laboratory conditions [4]. Although conventional PCR-based techniques allow for rapid detection of pathogens, they require the design of bulk primers based on the known sequences of pathogenic microorganisms. Therefore, the PCR-based techniques are limited to identifying only known or suspected pathogens rather than unknown ones [4–6]. Compared with the routine techniques, metagenomic sequencing (MGS) which is developed based on second-generation sequencing can be used to detect the pathogens with only a small amount of sample and free to microbial culture. It is potentially more suitable and valuable for diagnosing ocular infections, where sample availability is often limited. Moreover, MGS can identify ocular pathogens, including unknown or rare microorganisms.

In recent years, MGS has been applied to diagnose the pathogens of infectious uveitis, and it has greatly improved the efficiency and accuracy of clinical diagnosis of infectious uveitis [7]. For instance, Doan et al. employed unbiased MGS to analyze the intraocular fluid of uveitis patients and found that MGS could identify the pathogens of uveitis, such as fungi, parasites, and either DNA or RNA viruses, even rare viral pathogens like chronic intraocular rubella virus [8]. Similarly, Phadke et al. revealed that a patient living with HIV and relapsing uveitis was infected with human T-lymphotropic virus type 1 (HTLV-1) based on MGS [9]. In addition, Gonzales et al. reported a case of leptospirosis-associated uveitis confirmed by MGS after conventional culture and molecular assays failed to identify the pathogen [10]. Thus, MGS is a sensitive, unbiased, and efficient technique with great potential for diagnosing the causative pathogens of infectious uveitis.

In this study, MGS was used to analyze the aqueous humor samples of 20 patients (20 eyes) with infectious uveitis treated at the Affiliated Eye Hospital of Shandong University of Traditional Chinese Medicine between May 2020 and February 2021. The pathogens identified by MGS were validated using routine diagnostic techniques, including enzyme-linked immune sorbent assay (ELISA) and real-time quantitative PCR (qPCR). Finally, we discussed the advantages and clinical application of MGS in diagnosing infectious uveitis.

## Materials and methods

### General information

#### Subjects

The present study was approved by the Ethics Committee of the Affiliated Eye Hospital of Shandong University of Traditional Chinese Medicine and strictly adhered to the tenets of the Declaration of Helsinki. All subjects enrolled in the present study signed the informed consent. Twenty patients (30 eyes) who were suspected of infectious uveitis were consecutively enrolled between May 2020 and February 2021 at the Affiliated Eye Hospital of Shandong University of Traditional Chinese Medicine. They were designated S1 to S20. These patients were suspected infectious uveitis after undergoing comprehensive ophthalmologic examination, including slit-lamp examination, visual acuity testing, intraocular pressure measurement, ultra-wide field scanning laser ophthalmoscopy (SLO), B-scan ultrasonography, fluorescein fundus angiography (FFA), and other necessary systemic and auxiliary examinations (e.g., routine infectious disease screening, erythrocyte sedimentation rate [ESR], and C-Reactive protein [CRP]), though the specific pathogens were unknown. The demographic and ophthalmic characteristics including age, sex, best-corrected visual acuity (BCVA), intraocular pressure (IOP) keratic precipitates (KP), anterior chamber inflammation, and iris appearance were collected for each patient. Uveitis was classified and graded in accordance with the Standardization of Uveitis Nomenclature (SUN) [11].

#### Ophthalmologic examination

Among the 20 enrolled patients, two cases (10%) were with corneal edema and degeneration, three cases (15%) with pupil deformation and dilation, eight cases (40%) with sluggish pupillary light reflex, seven cases (35%) with partial posterior synechiae of the iris, two cases (10%) with iris depigmentation, and another two cases (10%) with snowball-like vitreous opacities.

## Methods

(1) Collection of aqueous humor: Aqueous humor samples of the patients were collected following the principles and procedures outlined in the *Expert Consensus for the Operating Procedures for Bacterial Examination of Ocular Infection (2019).* For the patients with monocular disease, the diseased eye was sampled. For the patients who suffered from binocular diseases, the more severely affected eye was selected. After rinsing the ocular surface with a sterile solution, 100–150 μl of aqueous humor was collected via anterior chamber paracentesis using a 27-gauge needle under slit-lamp guidance, carefully avoiding any contact with the iris or lens. The collected sample was immediately transferred to reaction tubes and divided into two parts: one for MGS, and the other for ELISA and qPCR testing. All samples were promptly stored in a − 80 °C freezer for further tests.

(2) Metagenomic sequencing.

### DNA extraction

Following the instructions of the TIANGEN DNA Mini kit (Tiangen Biotech Co., Ltd., Beijing), the extraction and purification of total DNA were conducted with 50 μl of aqueous humor. After determination of the concentration and quality (Sparkjade, China) in a 96-well plate (NEST Biotechnology, China), DNA was fragmented into 200–300 bp fragments using DNA nicking enzyme and stored in 200 μL DNA-free tubes (NEST Biotechnology, China) at −80 °C for subsequent analysis.

### Construction of DNA libraries and sequencing

DNA libraries were constructed according to the operation manual of QIAseq™ Ultralow Input Library Kit (180,495, QIAGEN, Germany). It mainly includes end alignment, adaptor ligation, and PCR amplification. After determination of the concentration and the quality of the samples (Sparkjade, China), libraries with different barcode tags were pooled and sequenced on the Illumina NextSeq 550 sequencing platform (Illumina, America) using 75-bp paired-end reads.

### Bioinformatic analysis

Raw sequencing reads were processed to remove adaptor sequences, low-quality reads, low-complexity reads, and short fragments, yielding high-quality reads. Human-derived sequences were further removed through mapping to the human reference genome with the SNAP software (v2.0.1). The remaining clean reads were then aligned to a comprehensive database of pathogenic microbial genomes using the Burrow-Wheeler Alignment (BWA) software (v0.7.17) and achieving a microbial composition profile. The microbial composition was further taxonomically classified into bacterial, viral, fungal, or parasitic categories with the Kraken method [12]. Subsequently, putative pathogens were identified based on similar criteria outlined by Doan et al. [8], including (1) Reads per million (RMP) values of candidate organisms should be greater than 50.0 in which the uniquely mapped reads were considered. (2) The candidate organisms should be reported to be potentially pathogenic in the clinical studies of uveitis [8].

### Pathogenic detection with qPCR and ELISA

Pathogen detection was further validated using qPCR and ELISA. Here, we only focused on the common pathogens of uveitis, including human herpes simplex virus 1 (HSV-1), human herpes simplex virus 2 (HSV-2), varicella zoster virus 3 (VZV), cytomegalovirus 5 (CMV), Epstein Barr virus, Epstein Barr virus 4 (EBV), and toxocara. Regarding ELISA, specific IgGs were detected using the SERION ELISA classic kit (Virion\Serion, Germany) with 50 μl of aqueous humor. Meanwhile, qPCR was performed using a PCR assay kit (BioGerm Medical Biotechnology, China) on an ABI Prism 7500 sequence detection system (Applied Biosystems, USA) with 50 μl of aqueous humor. The primer sequences used for qPCR were synthesized according to the reference [13] and are listed in Table S1. Both ELISA and qPCR were carried out for viral pathogen detection. However, only ELISA was employed to identify the *Toxocara* due to the limited aqueous humor for most patients.

## Results

### Characteristics of the enrolled patients

A total of 20 patients were enrolled in this study (Table 1). Ages ranged from 11 to 71 years, with an average of 38.35 ± 16.22 years old. The patients included seven cases of the right eye, three cases of the left eye, and ten cases of both eyes. The best-corrected visual acuity (BCVA) varied from HM/20 cm to 1.0, while intraocular pressure (IOP) ranged between 9.5 mmHg and 35.0 mmHg. Location analysis of uveitis revealed eight cases of anterior uveitis, two cases of intermediate uveitis, three cases of posterior uveitis, and 7 cases of panuveitis. Notably, patient S05 had a history of hematopoietic stem cell transplantation, and patient S07 had a history of HIV infection.

**Table 1:** Patient characteristics

Subject	Age	Gender^a^	Eye involvement^b^	BCVA^c^	IOP (mmHg)^d^	Location of uveitis^e^	Time to recent onset^f^	Keratic precipitates	Grades of anterior chamber cells and flare^g^	Appearance of iris, pupil and retina	Other symptoms
S01	29	F	Binocular (L)	1.00	20.7	IU	4 months	None	1 + , 1 +	None	Vitreous opacity
S02	39	M	Right	FC/50 cm	32.8	AU	15 days	Suet-like, pigmented	2 + , 2 +	Iris local atrophy	None
S03	26	M	Right	HM/20 cm	19.7	AU	20 days, recurrent	Dust-like, gray	3 + , 3 +	Iris segmental atrophy	Concurrent cataract
S04	31	M	Binocular (R)	FC/20 cm	20.7	Panuveitis	2 months, recurrent	Suet-like, pigmented	2 + , 2 +	Retinal hemorrhage	None
S05	33	M	Binocular (R)	0.04	13.0	Panuveitis	3 months, recurrent	Suet-like, gray	1 + , 2 +	Iris segmental atrophy, distorted pupil, retinal hemorrhage	None
S06	13	M	Binocular (R)	0.80	16.0	Panuveitis	2 months	Dust-like, gray	1 + , 1 +	None	None
S07	49	M	Left	1.00	13.5	Panuveitis	10 days	Suet-like, gray	1 + , 1 +	Retinal hemorrhage	None
S08	60	F	Right	0.30	11.0	Panuveitis	7 days	Dust-like	2 + , 2 +	Retinal edema	None
S09	47	M	Left	0.50	15.0	IU	7 days, recurrent	Dust-like, gray	None	None	Vitreous opacity
S10	51	F	Binocular (L)	0.08	15.5	Panuveitis	2 months	Dust-like, gray	1 + , 1 +	Retinal degeneration	None
S11	38	F	Binocular (R)	1.00	15.7	AU	2 days	Dust-like	1 + , 1 +	Iris local atrophy, posterior synechiae	None
S12	16	F	Binocular (L)	HM/30 cm	17.0	AU	7 days, recurrent	Suet-like, gray	Unclear	Iris local atrophy, anterior synechiae, distorted pupil	Corneal edema
S13	42	F	Right	0.15	15.7	Panuveitis	2 months	Dust-like, white	1 + , 1 +	Retinal annular proliferation	None
S14	56	M	Right	FC/30 cm	29.5	AU	1 years	Pigmented	1 + , 1 +	Iris patchy depigmentation, anterior synechiae, distorted pupil	None
S15	71	F	Binocular (R)	0.02	15.3	AU	2 months	Dust-like, gray	1 + , 1 +	None	Concurrent cataract
S16	57	F	Left	1.00	9.5	AU	10 days	Suet-like	1 + , 1 +	Iris patchy depigmentation	None
S17	29	F	Right	1.00	12.5	PU	10 months	None	None	None	None
S18	42	M	Binocular (L)	0.50	16.0	PU	1 days	None	None	None	None
S19	11	M	Right	0.10	35.0	AU	2 months	Suet-like	3 + , 3 +	Iris local atrophy, distorted pupil	Secondary glaucoma
S20	27	F	Binocular (R)	1.00	13.0	PU	1 months	None	None	Retinal nodules	None

^a^Gender of patients. F represents female; M represents male

^b^Eye involved in this study. For the patients with binocular diseases, the more severe eye is represented by the letter in the brackets, where L represents left, R represents right

^c^BCVA, best-corrected visual acuity

^d^IOP, intraocular pressure

^e^Location of uveitis. AU represents anterior uveitis; IU represents intermediate uveitis; PU represents posterior uveitis

^f^Time to recent onset means the time interval from recent onset to seeking medical attention

^g^The grades of anterior chamber cells and anterior chamber flare, which were graded according to the criteria outlined in the Standardization of Uveitis Nomenclature (SUN)

### Results of MGS

**Fig. 1:**
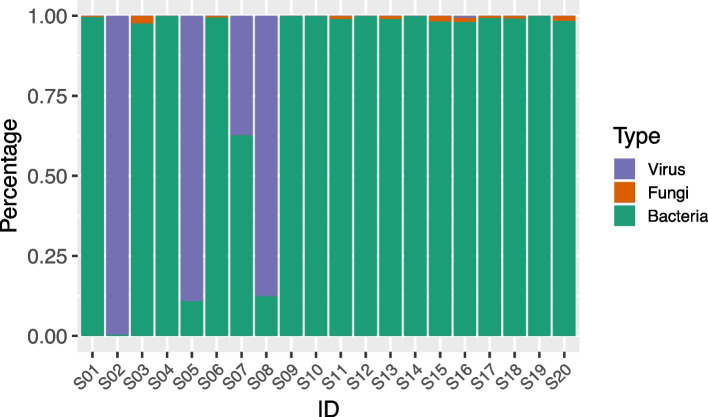
Distribution of relative scales of microbial sequences in 20 samples

After removing the low-quality sequencing and contaminated fragments, the cleaned metagenomic sequences of the samples were aligned to the reference pathogenic microbial genomes. Based on the taxonomic classification of microorganisms identified in the samples, bacteria were detected in all 20 samples (100%), fungi in 10 samples (50%), and viruses in 5 samples (25%) (Fig. 1). The relative abundance analysis revealed that bacteria predominated in most of the samples, accounting for an average relative abundance of 83.5 ± 33.75%). Fungi, when present, were detected at significantly lower relative abundances, averaging 1.11 ± 0.64% across 10 samples. Among the 5 samples (e.g., S02, S05, S07, S08, and S16) where viruses were detected, viruses often had the highest relative abundance except for sample S07 (relative abundance 37.22%) and S16 (relative abundance 0.56%).

Based on criteria including the RPM values derived from uniquely mapped fragments, the relative abundance of the suspected pathogens, the proportion within the same microbial category, and corroborating evidence from published studies, seven cases were identified as positive infections (Table 2), including bacteria, viruses, and fungi, while no parasitic infections were detected (Fig. 1). Among these seven confirmed cases, four involved viral infection, including two cases infected with cytomegalovirus (RPM: 891,276.18 and 372,222.22, respectively), one case infected with varicella-zoster virus (RPM: 875,621.89), and one case with both human herpes simplex virus and Epstein-Barr virus (RPM: 3,700.28 and 1,899.34, respectively). Two additional cases were involved with bacterial infections, including one case with *Pseudomonas aeruginosa* (RPM: 95,521.02) and one case with *Bacillus megaterium* (RPM: 15,306.12). Lastly, one case presented a mixed bacterial and viral infection, involving *Klebsiella pneumoniae* and varicella-zoster virus (RPM: 1,199.99 and 995,693.54, respectively). Notably, two cases infected with cytomegalovirus (S05 and S07) were consistent with their previous clinical history. However, no case of *Toxocara* infection was detected by MGS.

**Table 2:** Information of 7 positive cases identified by MGS

Subject	Location of uveitis	Microibial category	Pathogen	RPM based on unique reads	Proportion within the same category (%)	Relative abundance (%)	Validation by routine diagnostic methods^a^	References
S01	IU	Bacteria	Pseudomonas aeruginosa	95,521.02	9.58%	9.55%	-	[27, 34]
S02	AU	Bacteria	Klebsiella pneumoniae	1,199.99	28.57%	0.12%	VZV-IgG ( +), qPCR(-)	[30]
		Virus	Human alphaherpes virus 3	995,693.54	100.00%	99.57%		[35, 36]
S05	Panuveitis	Virus	Human betaherpes virus 5	891,276.18	100.00%	89.13%	CMV-IgG ( +), qPCR ( +)	[35, 36]
S07	Panuveitis	Virus	Human betaherpes virus 5	372,222.22	100.00%	37.22%	CMV-IgG ( +), qPCR ( +)	[35, 36]
S08	Panuveitis	Virus	Human alphaherpes virus 3	875,621.89	100.00%	87.56%	VZV-IgG ( +), qPCR ( +)	[35, 36]
S16	AU	Virus	Human alphaherpes virus 1	3,700.28	66.67%	0.37%	HSV-IgG ( +), qPCR(-)	[35, 36]
			Human gammaherpes virus 4	1,899.34	33.33%	0.19%		
S17	PU	Bacteria	Bacillus megaterium	15,306.12	1.54%	1.53%	-	[28]

a. Validation with ELISA or qPCR. Symbol “ + ” in the brackets represents a success validation by the method, while “-” indicates no testing was performed due to the limited sample

### Testing and verification

#### Antibody testing of pathogenic IgG

Antibody testing of pathogenic IgG

In the present study, specific IgG antibodies from common uveitis-related pathogens (including HSV, VZV, CMV, EBV, and *Toxocara*) were evaluated in 20 samples using ELISA. First of all, all five viral infections identified via MGS were confirmed by ELISA. Two cases (S02 and S08) with MGS-confirmed varicella-zoster virus infection demonstrated abnormally elevated VZV-IgG levels (684.00 U/ml and 842.93 U/ml, respectively). Meanwhile, another two cases with MGS-confirmed cytomegalovirus infection showed elevated CMV-IgG levels of 16.64 U/ml CMV-IgG in the S05 and 46.60 U/ml CMV-IgG in S07, exceeding the normal thresholds. Case S16 infected with both human herpes simplex virus and Epstein-Barr virus exhibited an elevated HSV-IgG level of 226.36 U/ml.

In addition, among the remaining 15 cases in which no viral pathogens were confirmed by MGS, ELISA revealed seven cases with abnormally high viral IgG levels, suggesting potential infections (Table 3). These included two cases (S13 and S18) with elevated CMV-IgG, another two cases with abnormally increased HSV-IgG, one case (S10) with elevated levels of both CMV-IgG and HSV-IgG, and two cases (S04 and S11) with elevated levels of VZV-IgG, CMV-IgG, and HSV-IgG. Furthermore, two cases were detected with *Toxocara* by ELISA (Table 3).

**Table 3:** ELISA results for 15 negative cases in MGS

Subject	Location of uveitis	Viral pathogens with ELISA	Toxocara with ELISA
S03	AU	-	-
S04	Panuveitis	VZV-IgG (31.98 U/ml), CMV-IgG (27.61 U/ml), HSV-IgG (148.53 U/ml)	-
S06	Panuveitis	HSV-IgG (9.03 U/ml)	-
S09	IU	HSV-IgG (24.06 U/ml)	-
S10	Panuveitis	CMV-IgG (34.47 U/ml), HSV-IgG (24.06 U/ml)	-
S11	AU	VZV-IgG (33.83 U/ml), CMV-IgG (295.61 U/ml), HSV-IgG (151.32 U/ml)	-
S12	AU	-	-
S13	Panuveitis	CMV-IgG (15.21 U/ml)	-
S14	AU	-	-
S15	AU	-	-
S18	PU	CMV-IgG (2.24 U/ml)	toxocara-IgG (15.39 U/ml)
S19	AU	-	toxocara-IgG (14.75 U/ml)
S20	PU	-	-

#### Validation of qPCR

qPCR has widely been used for detecting ocular pathogens, such as HSV, VZV, and CMV [14, 15]. Due to the limited volume of aqueous humor from the patients, qPCR testing was restricted to three of the cases with suspected viral infections identified by MGS, including two cases with cytomegalovirus (S05 and S07) and one case with varicella-zoster virus (S08). qPCR results confirmed the presence of cytomegalovirus-positive in S05 and S07, with the relative quantitative values 6.83E + 04 copies/ml and 3.69E + 03 copies/ml, respectively. Moreover, qPCR validated the varicella-zoster virus infection in S08, with relative quantitative values of 7.23E + 05 copies/ml. These findings were completely consistent with the MGS results.

## Discussion

### Advantages of MGS in diagnosing ophthalmic infectious diseases

Infectious uveitis is the inflammation of the uvea tract (including the iris, ciliary body, and choroid) caused by the infection of pathogens, and often progresses rapidly. If without timely and effective treatment, the condition may lead to severe complications such as anterior chamber hypopyon, panophthalmitis, and even blindness. Therefore, the timely and accurate identification of the causative pathogens for patients with infectious uveitis is critical for guiding effective clinical treatment. Due to the delicate and intricate anatomy of the eye, obtaining sufficient aqueous humor can be challenging. Traditional diagnostic methods, which rely on the cultivation of pathogens, are often limited in their scope and sensitivity, making comprehensive pathogen detection infeasible. These methods are additionally limited by their time-consuming process, incomplete diagnostic coverage, and high false-negative rates, all of which compromise their reproducibility and clinical utility [16–20]. By contrast, MGS offers a transformative approach. Using only a small volume of sample, MGS enables a comprehensive and unbiased analysis of the ocular microbiome, allowing the identification of both known and unknown pathogens with detailed taxonomic resolution.

Techniques such as ELISA and qPCR can detect specific pathogens with high sensitivity and accuracy. However, these techniques are inherently target-based and thus limited to known pathogens [4–6]. In contrast, MGS can operate without prior knowledge of pathogens, offering significant advantages in diagnosing uveitis caused by unknown or rare pathogens, Its broader detection capability facilitates the rapid identification of causative pathogens, enabling clinicians to tailor treatment strategies correctly [7].

### Common pathogens of infectious uveitis

The pathogens of infectious uveitis are highly diverse, with viral pathogens being the most prevalent. These include human herpes simplex virus (HSV-1 and HSV-2), VZV, CMV, EBV, and other viral pathogens [21]. Other causative agents include bacterial pathogens (e.g., *Mycobacterium tuberculosis* and *Staphylococcus aureus*), fungal pathogens (e.g., *Candida albicans* and *Cryptococcus neoformans*), or parasites (e.g., *Toxoplasma*) [22]. Notably, a large proportion of uveitis cases remain etiologically unresolved. The statistical analysis of one published study showed that the causative pathogens were successfully identified in only approximately 17% of infectious uveitis cases [23].

In the present study, we used MGS to detect the intraocular fluid samples from 20 patients with infectious uveitis, resulting in the identification of seven positive cases. Among them, five cases were infected with viral pathogens: two CMV infections (S05, S07), two VZV infections (S02, S08), and one co-effection with HSV-1 and EBV (S16). All identified viral pathogens belonged to the herpesvirus family, commonly associated with anterior or posterior uveitis, often accompanied by Fuchs syndrome or Posner–Schlossman syndrome [24, 25].

In addition, three cases were attributed to bacterial pathogens, including one case infected with *Pseudomonas aeruginosa* (S01), one case with *Bacillus megaterium* (S17), and one case involving *Klebsiella pneumoniae* co-infected with VZV (S02). Case S01 showed a high relative abundance of *Pseudomonas aeruginosa* (9.55%). The infection of Pseudomonas aeruginosa is known to usually lead to scleritis and is often accompanied by anterior uveitis [26, 27]. Similarly, *Bacillus megaterium* exhibited the highest relative abundance (1.53%) among the potential pathogens in case S17, which has been previously reported to be associated with uveitis [28]. Although case S02 was confirmed to be infected with VZV via both MGS and ELISA, it also showed a high relative abundance of *Klebsiella pneumoniae* (28.57% in the same category) according to the result of MGS. As an important opportunistic pathogen, *Klebsiella pneumoniae* is generally associated with systemic infections, such as pneumonia, urinary tract infections, and septicemia. Some studies have proposed its potential role in acute anterior uveitis, often accompanied by symptoms like eye redness, increased discharge, and endophthalmitis-like signs [29, 30]. However, patient S02 did not exhibit symptoms of acute uveitis, suggesting that *Klebsiella pneumoniae* was not the co-causative agent in this case.

### Limitations of MGS

Firstly, MGS cannot completely replace the conventional technologies at present. MGS identified five viral infections which were confirmed by ELISA or qPCR. However, ELISA detected elevated viral IgG in seven cases and Toxocara-IgG in two cases among the 15 MGS-negative cases, suggesting infections not captured by MGS. Similar to our results, a previous study analyzed 31 cases of anterior uveitis and found more virus infections (19 and 5 cases, respectively) detected by ELISA and qPCR than by MGS (3 cases) [31]. These observations underscore the complementary role of routinely targeted techniques (e.g., ELISA and qPCR) alongside MGS in clinical diagnosis, particularly for pathogens with low abundance or requiring serological confirmation.

Secondly, a mixed sequencing library of DNA and cDNA is required if RNA viruses need to be detected simultaneously by MGS. RNA viruses are an important category of pathogens associated with uveitis, such as Rubella virus (RV), a recognized causative agent of Fuchs uveitis [32]. Detecting RNA viruses with MGS relies on the extraction of total RNA and further reverse transcription to synthesize cDNA. However, due to the limited sample volume, we only extracted total DNA and constructed the libraries exclusively from DNA. As a result, MGS analysis in this study was limited to detecting DNA-based pathogens.

Lastly, the sensitivity and specificity of pathogen detection using MGS can be influenced by several critical factors, including sequencing depth, pathogen load, proportion of host-derived background, and potential contamination during the assay [33]. In this study, the limited sensitivity of MGS may be attributed to the low pathogen load in the small sample volume and insufficient sequencing depth. Hence, more investigations focusing on these influencing factors are essential to enhance the detection capabilities of MGS.

## Conclusion

We used MGS to identify the pathogens for patients with infectious uveitis and validated the results with routinely targeted technologies. The results highlight the potential of MGS in diagnosing infectious uveitis by identifying a broad range of pathogens, including viruses, bacteria, fungi, and previously unrecognized agents. While MGS cannot completely replace the traditional techniques, its comprehensive and unbiased detection capabilities make it a valuable tool for advancing the diagnostic landscape of infectious uveitis. Improvement of sensitivity and specificity is necessary for further promotion of MGS in ophthalmic diagnosis.

## Supplementary Information

Supplementary Material 1.

## Data Availability

All the data pertaining to the cases are available with the corresponding author.
