# Supplementary material for: Preliminary exploration of metagenomic sequencing for pathogenic identification in infectious uveitis
Source: J Ophthalmic Inflamm Infect. 2024 Dec 31;14:70. doi: 10.1186/s12348-024-00449-3 (PMC11688260; doi:10.1186/s12348-024-00449-3)
Supplement: Supplementary file 1 — Supplementary Material 1. [file 12348_2024_449_MOESM1_ESM.docx]

**Supplementary materials**

Table S1. The primer sequences used for qPCR tests.

Table S1. The primer sequences used for qPCR tests.

| Virus | Sequence of primer |
| --- | --- |
| HSV-1 | ATACCGACCACACCGACGA |
|  | ACAACTCCCTAACCCCTGCT |
| HSV-2 | TTCCCCCGTGGCTCAATATT |
|  | ACGCGCCGGGGCAGGTCT |
| VZV | GGCGGAACTTTCGTAACCAA |
|  | CCCCATTAAACAGGTCAACAAAA |
| EBV | CCAAGAAGGTGGCCCAGA |
|  | CCTGCCTCCATCACCCTG |
| CMV | TCGCGCCCGAAGAGG |
|  | CGGCCGGATTGTGGATT |
